# Supplementary material for: Pathophysiological Significance of Neutrophilic Transfer RNA-Derived Small RNAs in Asymptomatic Moyamoya Disease
Source: Cells. 2021 May 1;10(5):1086. doi: 10.3390/cells10051086 (PMC8147334; doi:10.3390/cells10051086)

2D Structure	Local AU	Position

```
881                        8mer-1a          910
5'-ttgcatgtgtgcacaaAGATGCATGTGGGa-3' utr
                   |||: ||||||||
3'-uccuuaggauuggcgaUCUG-GUACACCCu-5' tRF
                 16151413  7 6 5 4 3 2
               3' Pairing     Seed
```

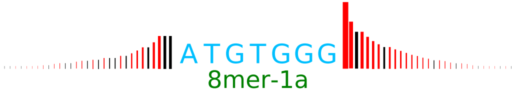

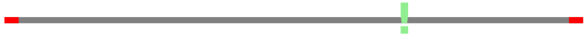

Supplement: Supplementary file 1 [file cells-10-01086-s001.zip › cells-1149161 3rd supplementary materials/Data Supplements/Data Supplement V.zip!/Data supplement 6-mRNAs targets of tsRNAs/tsRNA-Glu-TTC-010/Diagrams/tRF-Glu-TTC-010_vs_ENST00000328823(WBP2NL).pdf]
